# Lack of genetic support for shared aetiology of Coronary Artery Disease and Late-onset Alzheimer’s disease

**DOI:** 10.1038/s41598-018-25460-2

**Published:** 2018-05-08

**Authors:** Christopher Grace, Robert Clarke, Anuj Goel, Martin Farrall, Hugh Watkins, Jemma C. Hopewell

**Affiliations:** 1 Division of Cardiovascular Medicine, Radcliffe Department of Medicine, University of Oxford, Oxford, UK; 2 Wellcome Centre for Human Genetics, University of Oxford, Oxford, UK; 3 Clinical Trial Service Unit and Epidemiological Studies Unit, Nuffield Department of Population Health, University of Oxford, Oxford, UK

## Abstract

Epidemiological studies suggest a positive association between coronary artery disease (CAD) and late-onset Alzheimer’s disease (LOAD). This large-scale genetic study brings together ‘big data’ resources to examine the causal impact of genetic determinants of CAD on risk of LOAD. A two-sample Mendelian randomization approach was adopted to estimate the causal effect of CAD on risk of LOAD using summary data from 60,801 CAD cases from CARDIoGRAM*plus*C4D and 17,008 LOAD cases from the IGAP Consortium. Additional analyses assessed the independent relevance of genetic associations at the *APOE* locus for both CAD and LOAD. Higher genetically determined risk of CAD was associated with a slightly higher risk of LOAD (Odds Ratio (OR) per log-odds unit of CAD [95% CI]: 1.07 [1.01–1.15]; p = 0.027). However, after exclusion of the *APOE* locus, the estimate of the causal effect of CAD for LOAD was attenuated and no longer significant (OR 0.94 [0.88–1.01]; p = 0.072). This Mendelian randomization study indicates that the *APOE* locus is the chief determinant of shared genetic architecture between CAD and LOAD, and suggests a lack of causal relevance of CAD for risk of LOAD after exclusion of *APOE*.

## Introduction

Coronary artery disease (CAD), including myocardial infarction and angina, results from atherosclerosis of the underlying coronary arteries that causes obstruction to the blood supply of the heart^1,2^. Elevated levels of blood pressure and blood lipids, together with cigarette smoking and diabetes are established major risk factors for CAD^3^. However, CAD is a complex disease involving both environmental and genetic causes, with a heritability of 40–60%^4^. Genome-wide association studies (GWAS) have identified multiple genetic variants, but each has been associated with only a modest effect on risk of CAD. The CARDIoGRAM*plus*C4D meta-analysis, involving 60 801 CAD cases and 123 504 controls, identified a total of 57 variants that were associated with CAD^5^. Few of these variants encoded established risk factors and the effect sizes of these variants were small (OR 1.03–1.37), but together accounted for 13.3% of CAD heritability^5^.

Dementia is a clinical syndrome characterized by memory loss, difficulties in cognitive function, problem solving or language that affects a high proportion of older people. Alzheimer’s disease (AD) and vascular dementia are the two most common causes of dementia and despite a substantial overlap of their clinical presentation each have distinct neuropathological features^6^. AD pathology is characterized by extracellular amyloid plaques and intracellular neurofibrillary tangles that precede the onset of clinical symptoms by 1–2 decades^7^. By contrast, vascular dementia is characterized by micro infarcts and perivascular hyalinosis^8^. Late onset Alzheimer’s disease (LOAD) is diagnosed by clinical criteria, including insidious onset and progressive impairment of both memory and some other domains of cognitive function in the absence of motor, sensory or coordination deficits in individuals aged over 65 years^9^.

LOAD has a multifactorial inheritance (with a heritability of 60%)^10^, the most recent GWAS identified 21 variants that were significantly associated with LOAD^11^. The *APOE* locus is the most important locus for LOAD; with individuals with two copies of the ε4 allele (ε4/ε4) having a 15-fold higher risk (OR [95% CI]: 14.9 [10.8–20.6]) and individuals with one copy of the ε4 allele (ε3/ε 4) having a 3-fold higher risk of LOAD (OR [95% CI]: 3.2 [2.8–3.8]) compared with individuals with two copies of the *APOE*-ε3 allele (ε3/ε3)^12^. In contrast, carriers of the *APOE*-ε2 allele had a 40% lower risk (ε2/ε2 OR [95% CI]: 0.6 [0.2–2.0]) and (ε2/ε3 OR [95% CI]: 0.6 [0.5–0.8]) than individuals with two copies of the *APOE*-ε3 (ε3/ε3)^12^. Furthermore, the *APOE*-ε2 (rs7412) has been associated with LDL cholesterol concentrations (effect size [SD units, per C allele] [95% CI]: 0.59 [0.57–0.61]; p = 1.24 × 10^−652^)^13^, and rs4420638 (D′ = 1 with rs7412) was associated with a 10% higher risk of CAD (OR [95% CI]: 1.10 [1.07–1.13])^5^.

Observational studies have implicated atherosclerosis and cardiovascular risk factors in the initiation and progression of dementia, including AD^14–18^. The Rotterdam study reported a 3-fold higher risk of AD associated with the presence of carotid atherosclerosis (OR [95% CI]: 3.00 [1.50–6.00]; p = 0.001)^14^. Individuals with both carotid atherosclerosis and the *APOE* ε4 allele had a 4-fold higher risk of AD (OR [95% CI]: 3.90 [1.60–9.60]) than individuals without either risk factor.

Several studies have also reported that clinically manifest cardiovascular disease was associated with a higher risk of LOAD^16–18^. However, since LOAD has a very long latency period, such studies have been constrained by confounding, reverse causality bias and diagnostic misclassification. Mendelian randomization (MR) studies afford the potential to elucidate the causal relevance of lifelong differences in exposures with disease outcomes that are independent of confounding and reverse causation^19,20^. Moreover, MR studies have been successful in enhancing our understanding of the causal risk factors for cardiovascular diseases^21^.

The aim of the present study was to examine the causal relevance of CAD on risk of LOAD, and explore their shared genetic architecture. The objectives of this study were: (i) to assess the impact of genetic determinants of CAD on risk of LOAD; and (ii) to assess the independent relevance of genetic associations at the *APOE* locus for both CAD and LOAD.

## Methods

### Participating cohorts

The CARDIoGRAM*plus*C4D^5^ summary statistics were derived from a 1000 Genomes-based meta-analysis of 48 studies, involving 60 801 CAD cases and 123 504 controls. Diagnosis of CAD included evidence of myocardial infarction, chronic stable angina with a revascularisation procedure or a coronary stenosis >50% and CAD cases had a mean age of approximately 60 years^5^. The International Genomics of Alzheimer’s Project (IGAP)^11^ summary statistics were derived from a 1000 Genomes-based meta-analysis of LOAD cases among European individuals, involving 17 008 LOAD cases and 37 154 controls. Diagnosis of LOAD followed assessment by a neurologist and LOAD cases had a mean age of approximately 74 years^11^.

This study presents a new analysis of anonymised summary data from previously published meta-analyses in which each of the individual studies had ethics approval by the relevant institutions where participants (or, for those with substantial cognitive impairment, from an appropriate proxy), provided written informed consent^5,11^.

### Selection of variants

Among the 57 genome-wide significant variants identified in the CARDIoGRAM*plus*C4D meta-analysis, 52 genetic variants were selected for the present study after exclusions^5^. Variants with recessive associations (n = 2) and for which a suitable proxy was also unavailable (r^2^ ≥ 0.80) in IGAP (n = 3), were excluded from the analysis^11^ (eTable 1). Summary estimates (per effect allele) were extracted from the CARDIoGRAM*plus*C4D^5^ meta-analysis for CAD and from the IGAP^11^ meta-analysis for LOAD (eTables 2 and 3).

A sensitivity analysis including 190 genetic variants of the 214 (including 9 with a minor allele frequency <0.05) with a 5% False Discovery Rate (FDR) identified in the CARDIoGRAM*plus*C4D meta-analysis was performed^22^. A total of 24 variants were excluded due to the absence of a suitable proxy (i.e. no variant with r^2^ ≥ 0.80).

### Mendelian randomization analysis

The effect of CAD (risk phenotype) on LOAD (outcome phenotype) was analysed by looking at the impact of each genetic marker’s effect size for CAD on its effect size for LOAD. Effects of individual variants are given per copy of the effect allele unless otherwise stated. This was assessed by calculating the ratio of LOAD effect size/CAD effect size for each of the 52 variants, and combining them using a fixed effect meta-analysis model to estimate the causal effect^23^. The Cochran Q statistic was used to assess heterogeneity in risk estimates between the variants in the fixed effect meta-analysis. An online database (PhenoScanner)^24^ was used to identify multiple phenotypes associated with individual genetic variants to investigate potential pleiotropy. Sensitivity analyses using Egger regression MR^25^ were performed, a method that allows for invalid instrumental variables due to pleiotropy, using the MR-BASE R package^26^.

### Cross-trait LD score regression

The genetic correlation of effect statistics for CAD and for LOAD was estimated by cross-trait LD score regression^27^ using a total of 5,403,795 variants that were studied in both the CARDIoGRAM*plus*C4D and IGAP meta-analyses. This method estimates the genetic correlation between the two traits using GWAS summary statistics and is unbiased by any overlap of participants in both study populations^27^.

### Scan for shared genetic determinants at the APOE locus

Shared genetic determinants for LOAD and CAD at the *APOE* locus were investigated using a recently developed approach known as gwas-pw^28^. This method uses a statistical model to estimate the posterior probability that a genomic region adheres to four separate models. Models 1 and 2 are used to test whether a locus contains a genetic variant for only one of the two phenotypes. Models 3 and 4 are used to test whether a genetic variant exists for both phenotypes within the locus. More specifically, Model 3 assesses whether the same genetic variant influences both phenotypes, whilst Model 4 assesses whether the two phenotypes are influenced by separate genetic variants within a locus.

### Data availability

The datasets analysed during the current study are publically available in the CARDIoGRAM*plus*C4D^5^ (CAD) repository http://www.cardiogramplusc4d.org and the IGAP^11^ (LOAD) repository http://web.pasteur-lille.fr/en/recherche/u744/igap/igap_download.php.

## Results

### Mendelian randomization analyses

**Figure 1:**
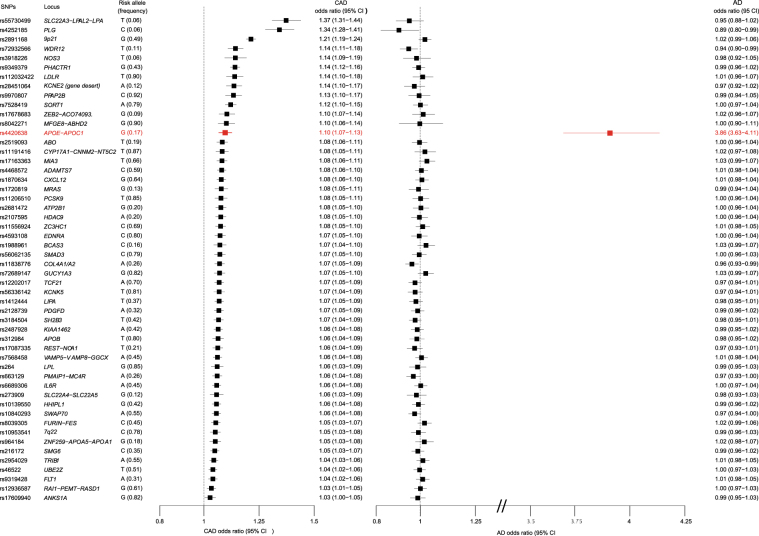
Forest plot of variants included in the Mendelian randomization analysis of the causal relevance of CAD for LOAD. Forest plot of odds ratios (OR) for the 52 variants used in the analysis with CAD and LOAD. Variants are reported for the increasing CAD risk allele (i.e. CAD ORs are >1). The *APOE* locus is marked in red.

Figure 1 shows the associations of the 52 variants that were used to estimate the causal effect. For each genome-wide significant variant, the odds ratio (OR) and 95% confidence intervals (CI) of the summary statistics in the CARDIoGRAM*plus*C4D and IGAP meta-analyses are presented. The CAD odds ratios are presented in descending order of strength of their association with CAD and indicate that the *APOE* rs4420638 genetic variant was the sole variant significantly associated with LOAD.

Table 1 shows the causal effect estimates on LOAD after combining information across all 52 CAD variants. The results indicate a nominally significant causal association, consistent with a higher risk of CAD being associated with a 7% higher risk of LOAD (OR 1.07 for LOAD per log odds unit of CAD [1.01–1.15]; p = 0.027). However, there was significant heterogeneity between the causal effects for each of the variants included in the analysis (p < 2.2 × 10^−308^). After removal of the single outlying variant, rs4420638 at the *APOE* locus, there was no remaining heterogeneity (p = 0.351). Furthermore, after removal of the *APOE* variant, there was no longer any significant causal association of CAD with LOAD (OR 0.94 for LOAD per log odds unit of CAD [0.88–1.01]; p = 0.072). The causal estimate (based on the 52 CAD variants) from the Egger regression approach was not significant (p = 0.846), suggesting that the *APOE* variant may not be a valid instrumental variable due to pleiotropy at the *APOE* locus. Similar results were observed using data for the 190 CAD variants that were significantly associated with CAD at the 5% FDR threshold.

**Table 1:** Results of Mendelian randomization analysis for CAD on LOAD.

Risk Phenotype	Variant count	OR for LOAD[95% CI]	p*-*value	Cochran’s Q	df	p-value
CAD (GWS)	52	1.07 [1.01–1.15]	0.027	1868.13	51	<2.2 × 10^−308^
CAD (GWS) (Excluding *APOE*)	51	0.94 [0.88–1.01]	0.072	53.22	50	0.351
CAD (GWS) Egger MR	52	1.09 [0.47–2.54]	0.846	1868.13	51	<2.2 × 10^−308^
CAD (FDR)	190	1.05 [1.01–1,10]	0.024	2059.25	189	<2.2 × 10^−308^
CAD (FDR) (Excluding *APOE*)	189	0.99 [0.94–1.03]	0.553	247.40	188	0.002

Results of the analysis of summary statistics from CAD and LOAD. GWS is Genome Wide Significant (p ≥ 5 × 10^−8^); FDR is a false discovery rate of 5%. *APOE* refers to the rs4420638 variant within the *APOE* locus.

### Cross-trait LD score regression analysis

A cross-trait regression analysis indicated a non-significant genetic correlation between LOAD and CAD (genetic correlation [95% CI]: −0.02 [−0.20–0.16]; p = 0.84). These results were consistent with the MR analysis after exclusion of the *APOE* variant.

### APOE locus

**Figure 2:**
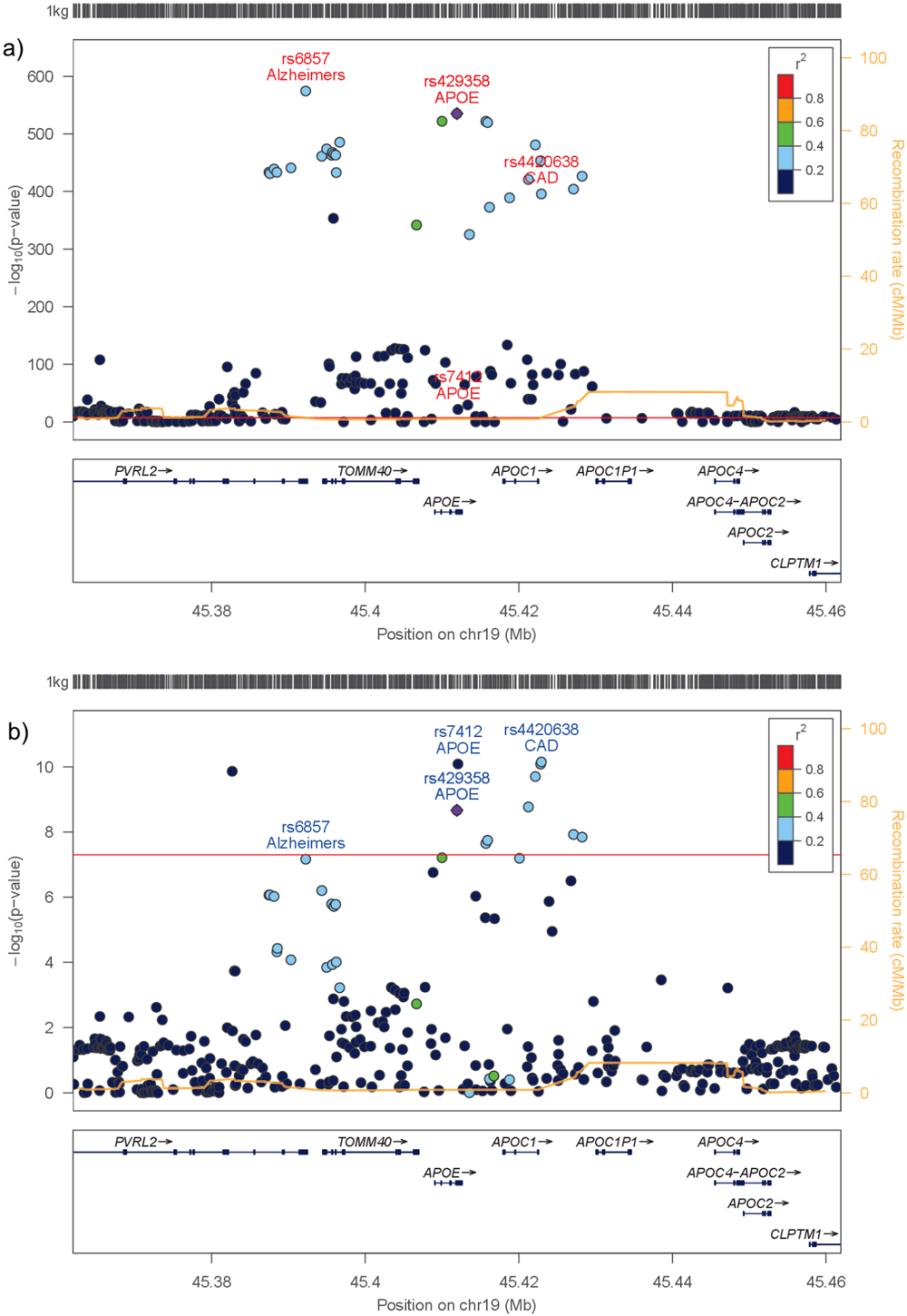
Signal plots of the *APOE* locus for LOAD and CAD. (**a**) LocusZoom^42^ signal plot of the *APOE* locus from the IGAP cohort. The strongest signal detected was for rs6857. Peak variant for CAD (rs4420638) and the *APOE* ε2 (rs7412) and ε4 (rs429358) variants are included. (**b**) Signal plot of the *APOE* locus from the CARDIoGRAM*plus*C4D meta-analysis. The strongest association signal detected was for rs4420638. Peak variant for LOAD (rs6857) and the *APOE* ε2 (rs7412) and ε4 (rs429358) variants are included. LD structure in both plots is in reference to the *APOE* ε4 (rs429358) variant.

Figure 2 shows a signal plot of the *APOE* loci for CAD and LOAD. The peak association signal for CAD was rs4420638 (OR [95% CI]: 1.10 [1.07–1.13]; p = 7.07 × 10^−11^; effect allele [EA] = G), and the peak signal for LOAD was rs6857 (OR [95% CI]: 3.19 [3.05–3.34]; p = 2.50 × 10^−575^; EA = G). The variants for the *APOE* ε2 allele (rs7412) and the *APOE* ε4 allele (rs429358) were also included in the figure. Table 2 shows the linkage disequilibrium (LD) structure (with D′ and r^2^) between the variants within the *APOE* region.

**Table 2:** Measures of linkage disequilibrium between variants at the *APOE* locus.

	rs6857	rs429358	rs7412	rs4420638
rs6857	1.00 (1.00)	0.64 (0.82)	0.01 (0.71)	0.42 (0.74)
rs429358		1.00 (1.00)	0.01 (0.68)	0.66 (0.95)
rs7412			1.00 (1.00)	0.02 (1.00)
rs4420638				1.00 (1.00)

Linkage disequilibrium estimates taken from European 1000 Genomes for variants within the *APOE* locus: r^2^ and D′ (in brackets). Variants shown are as follows: rs6857: Peak variant from the IGAP meta-analysis, rs429358: Variant comprising the *APOE* ε4 allele, rs7412: Variant comprising the *APOE* ε2 allele, rs4420638: Peak variant from the CARDIoGRAM*plus*C4D meta-analysis.

Investigation of the LD structure between the four variants, indicated that rs7412 (the *APOE* ε2 allele) had a weak r^2^ with the other variants (rs6857: 0.01, rs429358: 0.01 and rs4420638: 0.02), but moderate to high D′ (rs6857: 0.71, rs429358: 0.68 and rs4420638: 1.00) and with complete LD with rs4420638. Different LD observations for r^2^ and D′ reflect the differing allele frequencies between rs7412 and the other variants (eTables 4 and 5). The remaining variants were in moderate to high LD with each other for both r^2^ and D′ (Table 2 and eTable 6) (r^2^: 0.42–0.65; D′: 0.74–0.95). The peak variant for LOAD (rs6857) was in stronger LD with the *APOE*-ε4 variant rs429358 (D′: 0.82) than the *APOE*-ε2 variant rs7412 (D′: 0.71). The peak variant for CAD rs4420638 was in stronger LD with the *APOE*-ε2 variant rs7412 (D′: 1.00) than the *APOE*-ε4 variant rs429358 (D′: 0.95).

### PhenoScanner

The PhenoScanner database^24^ was searched to detect potential pleiotropy of the 52 variants included in the analysis (eTable 7). The results of the association p-values < 0.0012 (Bonferroni adjusted p < 0.05) are shown in eTable 7. Ten of the 52 variants were significantly associated with plasma LDL cholesterol concentrations, while *APOE* was the sole variant that was significantly associated with LOAD.

Analysis at the *APOE* locus (eTables 8–11), indicated that rs6857 (p = 5.12 × 10^−110^), rs4420638 (p = 1.51 × 10^−178^) and rs7412 (p = 1.24 × 10^−652^) were highly significantly associated with plasma LDL-cholesterol concentrations in the Global Lipids Genetics Consortium (GLGC)^13^. However, the *APOE*-ε4 variant (rs429358) was not tested in the GLGC study. In another LDL-cholesterol study in which rs429358 was tested^29^ the *APOE*-ε2 variant rs7412 (p = 5.54 × 10^−30^) was more significant than the *APOE*- ε4 variant rs429358 (p = 4.21 × 10^−10^). Likewise, rs4420638 (p = 8.80 × 10^−139^) was also significantly associated with plasma concentrations of C-reactive protein (CRP)^30^. The variants rs6857 (p = 1.06 × 10^−10^) and rs429358 (p = 5.45 × 10^−14^) were also significantly associated with cortical amyloid beta load^31^.

### Shared impact of APOE locus

The gwas-pw method^28^ was used to detect evidence of shared genetic determinants within the *APOE* locus (chr19:44,744,147-46,101,600) for CAD and LOAD. There was strong evidence for genetic variants influencing both phenotypes at the locus. Furthermore Model 4 (Posterior Probability: 0.90), which specifies separate genetic variants within the *APOE* locus influencing CAD and LOAD, had a higher posterior probability than Model 3 (Posterior Probability: 0.10) which specified a shared genetic variant in the *APOE* locus.

## Discussion

### Disparate genetic architecture of CAD and LOAD

The present study investigated a shared genetic architecture between CAD and LOAD using large-scale GWAS meta-analyses for both diseases. The initial Mendelian randomization analysis suggested that a higher risk of CAD (per log odds unit) was also associated with a 7% higher risk of LOAD. However, there was significant heterogeneity between the causal effects of individual variants. This heterogeneity was entirely explained by a single variant (rs4420638) at the *APOE* locus. When the *APOE* variant was removed from the analysis, the causal effect on LOAD was completely attenuated and no longer significant. Thus, overall, genetic determinants associated with a higher risk of CAD were not significantly associated with LOAD after excluding variants at the *APOE* locus. In addition, the LD score regression analysis identified little or no genetic correlation between CAD and LOAD.

### APOE locus

The *APOE* locus was investigated in greater detail since the rs4420638 variant was strongly associated with both CAD (p = 7.07 × 10^−11^) and LOAD (p = 1.67 × 10^−396^). The gwas-pw analysis^28^, suggested that the traits were influenced by separate genetic variants within the *APOE* locus. This indicates that the influence of the *APOE* locus on both LOAD and CAD may be mediated through different mechanisms.

In the case of the *APOE* variants (rs7412, rs429358): association with LDL cholesterol, a stronger signal was detected in the *APOE*-ε2 variant (rs7412, p = 5.54 × 10^−30^)^29^ than in the *APOE*-ε4 variant (rs429358, p = 4.21 × 10^−10^)^29^, suggesting that the *APOE*-ε2 variant is strongly associated with LDL cholesterol pathways. In the case of amyloid beta load, the *APOE*-ε4 variant has been strongly associated with this phenotype (rs429358; OR not available, p = 5.45 × 10^−14^)^31^, suggesting that the *APOE*-ε4 effect may be mediated by amyloid beta pathways. However the *APOE*-ε2 variant (rs7412) was not present in the analysis of amyloid beta.

In the case of LOAD, the *APOE*-ε4 variant (rs429358, OR [95% CI]: 3.86 [3.66–4.07]; p = 6.70 × 10^−536^; EA = C) had a stronger effect than the *APOE*-ε2 variant (rs7412, OR [95% CI]: 1.47 [1.36–1.59]; p = 1.23 × 10^−22^; EA = C)^11^ suggesting that LOAD may be primarily associated with the *APOE*-ε4 variant: The *APOE*-ε4 (rs429358) variant is also the peak signal for cortical amyloid beta load^31^ suggesting that variants in *APOE* for LOAD may be primarily associated with amyloid beta pathways. In contrast, CAD had a similar strength of association with both the *APOE*-ε4 variant (rs429358, OR [95% CI]: 1.10 [1.06–1.13]; p = 2.17 × 10^−9^; EA = C) and *APOE*-ε2 variant (rs7412, OR [95% CI]: 1.15 [1.10–1.20]; p = 8.17 × 10^−11^; EA = C)^5^. The CAD peak variant (rs4420638) was in complete LD (by measures of D′) with the *APOE*-ε2 variant (rs7412) suggesting that variants in *APOE* for CAD may be primarily associated with LDL cholesterol pathways. Future analyses of individual participant data could permit exploration of the ε2/ε3/ε4 *APOE* haplotypes to further elucidate these relationships.

Exploration of potentially pleiotropic effects in each of the 52 variants for CAD (eTable 7) suggest that LDL cholesterol is unlikely to be involved in any shared biological pathways between LOAD and CAD, other than via the effects of *APOE*. Among the 10 CAD loci that were associated with significant differences in LDL-cholesterol concentrations, only one of these (*APOE*) was also associated with LOAD.

### Other MR analyses

Recently, several studies have conducted MR analyses of vascular risk factors with LOAD. Østergaard and colleagues^32^ reported that a genetically predicted 1-SD (15.4 mmHg) higher systolic blood pressure was associated with lower risk of AD (OR [95% CI]: 0.75 [0.62–0.91]; p = 3.4 × 10^−3^); and that a 1-SD (0.91 mmol/l) higher LDL cholesterol was associated with a higher risk of AD (OR [95% CI]: 2.31 [2.12–2.50]; p = 3.0 × 10^−87^); and that a 1-SD (0.41 mmol/l) higher HDL cholesterol levels was associated with lower risk of AD (OR [95% CI]: 0.75 [0.69–0.82]; p = 1.0 × 10^−11^). However, after removing the *APOE* locus (rs6857), the associations were no longer significant for LDL-cholesterol (OR [95% CI]: 1.07 [0.98–1.17]; p = 0.14) or HDL-cholesterol (OR [95% CI]: 1.01 [0.93–1.09]; p = 0.87).

Another study reported null associations between genetically-predicted body mass index with LOAD^33^. Likewise, variants encoding Type-2 diabetes were also unrelated to LOAD^34^.

The METASTROKE consortium^35^ examined the genetic association between ischemic stroke (IS) and LOAD using GREML^36^, and reported evidence of a shared genetic contribution of LOAD with small vessel stroke (genetic correlation [95% CI]: 0.37 [0.04–0.70]; p = 0.01). However, variants encoding large vessel stroke, which has a shared genetic architecture with CAD^37^, were unrelated to LOAD (genetic correlation [95% CI]: 0.00 [−0.22–0.22]; p = 0.49). Variants encoding cardioembolic stroke were also unrelated to LOAD (genetic correlation [95% CI]: 0.08 [−0.16–0.32]; p = 0.25)

### Limitations of the study

The present analysis was performed on 52 variants selected from the CARDIoGRAM*plus*C4D meta-analysis, at which a genome-wide significant signal (p ≤ 5 × 10^−8^) had been identified^5,38–41^. The results of this analysis were not materially altered when including 190 variants based on an FDR 5% threshold. Furthermore, results from LD score regression, examining information across the genome, also provide further support.

Results of an *post-hoc* power analysis are presented in eTable 12, which suggest that the analyses with genome wide significant and FDR 5% instrumental variables had ~80% power to identify ~10% effect on LOAD. Thus, later studies are unlikely to discover a material shared genetic architecture between CAD and LOAD.

The available evidence on the *APOE* locus indicated separate mechanisms by which this locus acts upon LOAD and CAD, which raises questions about the assumptions for MR analysis involving this locus. Further fine mapping studies of the *APOE* locus are needed to assess the genetic associations at these highly correlated variants. These studies could include populations with greater haplotype diversity to resolve tightly linked genetic signals that appear intractably interwoven in Europeans. However, we encountered difficulties with exploratory fine mapping studies of the *APOE* locus for LOAD, due to collinearity induced by the strong linkage disequilibrium between the variants in this locus.

The present study also had several limitations. Firstly, the study maybe constrained by selection bias due to differences in age. The average age of onset of CAD was approximately 60 years, while the average age of onset of LOAD cases was 74 years. Individuals predisposed to developing both LOAD and CAD may not have survived to old age, which may have underestimated any association with LOAD. Moreover, the diagnosis of probable LOAD excludes a prior history of cerebrovascular disease, so studies of LOAD may have reduced risk of overlap between CAD and LOAD. However, neuroimaging studies show that pathological changes in the brain precede the development of mild cognitive impairment and LOAD by 1–2 decades^7^.

Selection bias may also have influenced summary statistics between CAD and LOAD due to overlapping participants for each disease in some studies. However, the number of overlapping cases in AGES, Rotterdam and Framingham Heart studies is very limited (up to 1000 LOAD cases in IGAP could potentially overlap with CAD cases included in the CARDIoGRAM*plus*C4D study).

Another possible limitation is that since the CARDIoGRAM*plus*C4D data included some non-European samples, these could increase heterogeneity between the samples. However, the CARDIoGRAM*plus*C4D study reported that no heterogeneity between studies was observed at any of the genome wide significant variants apart from the 9p21 locus^5^.

The present report demonstrated a different genetic architecture of CAD and LOAD. While further studies are required to further elucidate links between cardiovascular disease risk factors and LOAD, additional MR studies of CAD are unlikely to be informative about the causes of LOAD.

## Conclusions

Analyses were performed to investigate whether CAD and LOAD have a shared genetic architecture and whether CAD is a causal risk factor for LOAD, given the findings of observational studies. However, the present study demonstrated that although genetic predisposition to CAD was significantly associated with LOAD, this association was entirely mediated through the *APOE* locus. After exclusion of the *APOE* locus, CAD variants were no longer significantly associated with LOAD. Additional fine mapping studies are needed to dissect the independent relevance of *APOE* for both CAD and LOAD.

## Electronic supplementary material

Supplementary Material
