# Supplementary material for: Lack of genetic support for shared aetiology of Coronary Artery Disease and Late-onset Alzheimer’s disease
Source: Sci Rep. 2018 May 8;8:7102. doi: 10.1038/s41598-018-25460-2 (PMC5940751; doi:10.1038/s41598-018-25460-2)
Supplement: Supplementary file 1 — Supplementary Material [file 41598_2018_25460_MOESM1_ESM.docx]

Supplementary Material

Lack of genetic support for shared aetiology of Coronary Artery Disease and Late-onset Alzheimer’s disease

Christopher Grace PhD* ^a,b^, Robert Clarke FRCP^c^, Anuj Goel MBBS^a,b^, Martin Farrall FRCPath^a,b^, Hugh Watkins FRCP^a,b^, Jemma C Hopewell PhD^c^

Affiliations:

^a^ Division of Cardiovascular Medicine, Radcliffe Department of Medicine, University of Oxford, Oxford, UK.

^b^ Wellcome Trust Centre for Human Genetics, University of Oxford, Oxford, UK.

^c^ Clinical Trial Service Unit and Epidemiological Studies Unit, Nuffield Department of Population Health, University of Oxford, Oxford, UK.

Online Supplementary Material

**Lack of genetic support for shared aetiology of Coronary Artery Disease and Late-onset Alzheimer’s disease**

| Table of contents | |  | Page |
| --- | --- | --- | --- |
| Membership of CARDIoGRAM*plus*C4D Consortium, International Genomics of Alzheimer’s Project (IGAP), and Genetic and Environmental Risk for Alzheimer’s disease (GERAD) | | | 2 |
| eTable 1 | List of variants or proxies for such variants used in the analysis | | 3 |
| eTable 2 | IGAP proxies for CARDIoGRAM*plus*C4D variants | | 4 |
| eTable 3 | LD matrix of instrumental variables for 52 variants for CAD | | 5 |
| eTable 4 | Allele frequency of alleles at the *APOE* locus, by *APOE* SNPs | | 6 |
| eTable 5 | Allele frequencies of additional alleles at the *APOE* locus, by *APOE* SNPs from CARDIoGRAM*plus*C4D | | 7 |
| eTable 6 | Association of variants in the *APOE* locus with CAD, LOAD and LDL cholesterol concentrations | | 8 |
| eTable 7 | Pleiotropy at CAD variants using a Phenoscanner analysis of relevant traits | | 9 |
| eTable 8 | Pleiotropic phenotypes at *APOE* peak variant rs6857 in IGAP | | 11 |
| eTable 9 | Pleiotropic phenotypes at *APOE* peak variant rs4420638 in CARDIoGRAM*plus*C4D | | 12 |
| eTable 10 | Pleiotropic phenotypes at the variant rs429358 (*APOE ε4)* in Phenoscanner analysis of relevant traits | | 13 |
| eTable 11 | Pleiotropic phenotypes at the variant rs7412 (*APOE ε2*) in Phenoscanner analysis of relevant traits | | 14 |
| eTable 12 | *Post-Hoc* power calculation for the GWS significant analysis and the FDR 214 analysis | | 15 |
| SUPPLEMENTARY REFERENCES* | | | 16 |
| *^*^Supplementary references for studies included in the Online Supplementary Material are displayed in curly brackets { } to distinguish these from the references used in the main manuscript that are displayed using square brackets [ ].* | | |  |

**CARDIoGRAM*plus*C4D Consortium membership**

Nikpay M, Goel A, Won HH, Hall LM, Willenborg C, Kanoni S, Saleheen D, Kyriakou T, Nelson CP, Hopewell JC, Webb TR, Zeng L, Dehghan A, Alver M, Armasu SM, Auro K, Bjonnes A, Chasman DI, Chen S, Ford I, Franceschini N, Gieger C, Grace C, Gustafsson S, Huang J, Hwang SJ, Kim YK, Kleber ME, Lau KW, Lu X, Lu Y, Lyytikäinen LP, Mihailov E, Morrison AC, Pervjakova N, Qu L, Rose LM, Salfati E, Saxena R, Scholz M, Smith AV, Tikkanen E, Uitterlinden A, Yang X, Zhang W, Zhao W, de Andrade M, de Vries PS, van Zuydam NR, Anand SS, Bertram L, Beutner F, Dedoussis G, Frossard P, Gauguier D, Goodall AH, Gottesman O, Haber M, Han BG, Huang J, Jalilzadeh S, Kessler T, König IR, Lannfelt L, Lieb W, Lind L, Lindgren CM, Lokki ML, Magnusson PK, Mallick NH, Mehra N, Meitinger T, Memon FU, Morris AP, Nieminen MS, Pedersen NL, Peters A, Rallidis LS, Rasheed A, Samuel M, Shah SH, Sinisalo J, Stirrups KE, Trompet S, Wang L, Zaman KS, Ardissino D, Boerwinkle E, Borecki IB, Bottinger EP, Buring JE, Chambers JC, Collins R, Cupples LA, Danesh J, Demuth I, Elosua R, Epstein SE, Esko T, Feitosa MF, Franco OH, Franzosi MG, Granger CB, Gu D, Gudnason V, Hall AS, Hamsten A, Harris TB, Hazen SL, Hengstenberg C, Hofman A, Ingelsson E, Iribarren C, Jukema JW, Karhunen PJ, Kim BJ, Kooner JS, Kullo IJ, Lehtimäki T, Loos RJ, Melander O, Metspalu A, März W, Palmer CN, Perola M, Quertermous T, Rader DJ, Ridker PM, Ripatti S, Roberts R, Salomaa V, Sanghera DK, Schwartz SM, Seedorf U, Stewart AF, Stott DJ, Thiery J, Zalloua PA, O'Donnell CJ, Reilly MP, Assimes TL, Thompson JR, Erdmann J, Clarke R, Watkins H, Kathiresan S, McPherson R, Deloukas P, Schunkert H, Samani NJ, Farrall M.

**IGAP Consortium membership**

Jun G, Ibrahim-Verbaas CA, Vronskaya M, Lambert JC, Chung J, Naj AC, Kunkle BW, Wang LS, Bis JC, Bellenguez C, Harold D, Lunetta KL, Destefano AL, Grenier-Boley B, Sims R, Beecham GW, Smith AV, Chouraki V, Hamilton-Nelson KL, Ikram MA, Fievet N, Denning N, Martin ER, Schmidt H, Kamatani Y, Dunstan ML, Valladares O, Laza AR, Zelenika D, Ramirez A, Foroud TM, Choi SH, Boland A, Becker T, Kukull WA, van der Lee SJ, Pasquier F, Cruchaga C, Beekly D, Fitzpatrick AL, Hanon O, Gill M, Barber R, Gudnason V, Campion D, Love S, Bennett DA, Amin N, Berr C, Tsolaki M, Buxbaum JD, Lopez OL, Deramecourt V, Fox NC, Cantwell LB, Tárraga L, Dufouil C, Hardy J, Crane PK, Eiriksdottir G, Hannequin D, Clarke R, Evans D, Mosley TH Jr, Letenneur L, Brayne C, Maier W, De Jager P, Emilsson V, Dartigues JF, Hampel H, Kamboh MI, de Bruijn RF, Tzourio C, Pastor P, Larson EB, Rotter JI, O'Donovan MC, Montine TJ, Nalls MA, Mead S, Reiman EM, Jonsson PV, Holmes C, St George-Hyslop PH, Boada M, Passmore P, Wendland JR, Schmidt R, Morgan K, Winslow AR, Powell JF, Carasquillo M, Younkin SG, Jakobsdóttir J, Kauwe JS, Wilhelmsen KC, Rujescu D, Nöthen MM, Hofman A, Jones L; IGAP Consortium., Haines JL, Psaty BM, Van Broeckhoven C, Holmans P, Launer LJ, Mayeux R, Lathrop M, Goate AM, Escott-Price V, Seshadri S, Pericak-Vance MA, Amouyel P, Williams J, van Duijn CM, Schellenberg GD, Farrer LA.

**GERAD Consortium membership**

Hollingworth P, Harold D, Sims R, Gerrish A, Lambert JC, Carrasquillo MM, Abraham R, Hamshere ML, Pahwa JS, Moskvina V, Dowzell K, Jones N, Stretton A, Thomas C, Richards A, Ivanov D, Widdowson C, Chapman J, Lovestone S, Powell J, Proitsi P, Lupton MK, Brayne C, Rubinsztein DC, Gill M, Lawlor B, Lynch A, Brown KS, Passmore PA, Craig D, McGuinness B, Todd S, Holmes C, Mann D, Smith AD, Beaumont H, Warden D, Wilcock G, Love S, Kehoe PG, Hooper NM, Vardy ER, Hardy J, Mead S, Fox NC, Rossor M, Collinge J, Maier W, Jessen F, Rüther E, Schürmann B, Heun R, Kölsch H, van den Bussche H, Heuser I, Kornhuber J, Wiltfang J, Dichgans M, Frölich L, Hampel H, Gallacher J, Hüll M, Rujescu D, Giegling I, Goate AM, Kauwe JS, Cruchaga C, Nowotny P, Morris JC, Mayo K, Sleegers K, Bettens K, Engelborghs S, De Deyn PP, Van Broeckhoven C, Livingston G, Bass NJ, Gurling H, McQuillin A, Gwilliam R, Deloukas P, Al-Chalabi A, Shaw CE, Tsolaki M, Singleton AB, Guerreiro R, Mühleisen TW, Nöthen MM, Moebus S, Jöckel KH, Klopp N, Wichmann HE, Pankratz VS, Sando SB, Aasly JO, Barcikowska M, Wszolek ZK, Dickson DW, Graff-Radford NR, Petersen RC; Alzheimer's Disease Neuroimaging Initiative., van Duijn CM, Breteler MM, Ikram MA, DeStefano AL, Fitzpatrick AL, Lopez O, Launer LJ, Seshadri S; CHARGE consortium., Berr C, Campion D, Epelbaum J, Dartigues JF, Tzourio C, Alpérovitch A, Lathrop M; EADI1 consortium., Feulner TM, Friedrich P, Riehle C, Krawczak M, Schreiber S, Mayhaus M, Nicolhaus S, Wagenpfeil S, Steinberg S, Stefansson H, Stefansson K, Snaedal J, Björnsson S, Jonsson PV, Chouraki V, Genier-Boley B, Hiltunen M, Soininen H, Combarros O, Zelenika D, Delepine M, Bullido MJ, Pasquier F, Mateo I, Frank-Garcia A, Porcellini E, Hanon O, Coto E, Alvarez V, Bosco P, Siciliano G, Mancuso M, Panza F, Solfrizzi V, Nacmias B, Sorbi S, Bossù P, Piccardi P, Arosio B, Annoni G, Seripa D, Pilotto A, Scarpini E, Galimberti D, Brice A, Hannequin D, Licastro F, Jones L, Holmans PA, Jonsson T, Riemenschneider M, Morgan K, Younkin SG, Owen MJ, O'Donovan M, Amouyel P, Williams J.

|  |
| --- |

**eTable 1:** List of variants or proxies for such variants used in the analysis

| **Variant** | **Status** | **Proxy** | **Known locus** |
| --- | --- | --- | --- |
| rs6903956* | Excluded | # | ADTRP-C6orf105 |
| rs10139550 | Included | # | *HHIPL1* |
| rs10953541 | Included | # | *7q22* |
| rs11191416 | Included | # | *CYP17A1-CNNM2-NT5C2* |
| rs11206510 | Included | # | *PCSK9* |
| rs11556924 | Included | # | *ZC3HC1* |
| rs11838776 | Included | # | *COL4A1/A2* |
| rs12202017 | Included | # | *TCF21** |
| rs12936587 | Included | # | *RAI1-PEMT-RASD1* |
| rs1412444 | Included | # | *LIPA* |
| rs17609940 | Included | # | *ANKS1A* |
| rs17678683 | Included | # | *ZEB2-ACO74093.1* |
| rs1870634 | Included | # | *CXCL12* |
| rs2107595 | Included | # | *HDAC9* |
| rs2128739 | Included | # | *PDGFD* |
| rs216172 | Included | # | *SMG6* |
| rs2487928 | Included | # | *KIAA1462* |
| rs2519093 | Included | # | *ABO* |
| rs264 | Included | # | *LPL* |
| rs2681472 | Included | # | *ATP2B1* |
| rs273909 | Included | # | *SLC22A4-SLC22A5* |
| rs28451064 | Included | # | *KCNE2 {gene desert}* |
| rs2891168 | Included | # | *9p21* |
| rs2954029 | Included | # | *TRIB1* |
| rs3184504 | Included | # | *SH2B3* |
| rs4252185 | Included | # | *PLG* |
| rs4420638*** | Included | rs41377151 | *APOE-APOC1* |
| rs4468572 | Included | # | *ADAMTS7* |
| rs4593108 | Included | # | *EDNRA* |
| rs46522 | Included | # | *UBE2Z* |
| rs55730499 | Included | # | *SLC22A3-LPAL2-LPA* |
| rs56336142 | Included | # | *KCNK5* |
| rs6689306 | Included | # | *IL6R* |
| rs72689147 | Included | # | *GUCY1A3* |
| rs7528419 | Included | # | *SORT1* |
| rs7568458 | Included | # | *VAMP5-VAMP8-GGCX* |
| rs9319428 | Included | # | *FLT1* |
| rs9349379 | Included | # | *PHACTR1* |
| rs964184 | Included | # | *ZNF259-APOA5-APOA1* |
| rs9970807 | Included | # | *PPAP2B* |
| chr2:44074126:D** | No Proxy | # | *ABCG5-ABCG8* |
| rs16986953** | No Proxy | # | *AK097927* |
| chr2:203828796:I | Proxy | rs72932566 | *WDR12* |
| chr2:21378433:D | Proxy | rs312984 | *APOB* |
| chr3:138099161:I | Proxy | rs1720819 | *MRAS* |
| rs17514846 | Proxy | rs8039305 | *FURIN-FES* |
| rs56289821 | Proxy | rs112032422 | *LDLR* |
| rs67180937 | Proxy | rs17163363 | *MIA3* |
| rs10840293 | Included | # | *SWAP70* |
| rs17087335 | Included | # | *REST-NOA1* |
| rs3918226 | Included | # | *NOS3* |
| rs56062135 | Included | # | *SMAD3* |
| rs663129 | Included | # | *PMAIP1-MC4R* |
| rs8042271 | Included | # | *MFGE8-ABHD2* |
| rs180803** | No Proxy | # | *POM121L9P-ADORA2A* |
| rs7212798 | Proxy | rs1988961 | *BCAS3* |
| rs11830157** | Recessive | # | *KSR2* |
| rs12976411** | Recessive | # | *ZNF507-LOC400684* |

*Variant (rs6903956) excluded from the analysis due to lack of significance in the CARDIoGRAM*plus*C4D meta-analysis (identified in a three-way GWAS in Chinese Han population) {26}.

** Variants excluded due to no suitable proxy or a recessive association model

***The *APOE* locus: The identifier of the rs4420638 SNP in the IGAP dataset is rs41377151.

**eTable 2:** IGAP proxies for CARDIoGRAM*plus*C4D variants

| **Lead Variant** | | | | | | | **Proxy Variant** | | | | | | | | | |
| --- | --- | --- | --- | --- | --- | --- | --- | --- | --- | --- | --- | --- | --- | --- | --- | --- |
| Chr | **Position  (bp)*** | **Variant** | **Alleles** | **Frequency** | **CAD OR (95% CI)** | **CAD  p-value** | **Position (bp)*** | **Variant** | **Alleles** | **Frequency** | **R2** | **Distance** | **CAD OR  (95% CI)** | **CAD  p-value** | **ALZ OR  (95% CI)** | **ALZ  p-value** |
| 2 | 203,828,796 | chr2:203828796:I | I/D | 0.0714 | 1.15 (1.11-1.18) | 2.15E-18 | 203,855,342 | rs72932566 | T/C | 0.0728 | 0.9818 | 26546 | 1.14 (1.11-1.18) | 8.58E-18 | 0.94 (0.9-0.99) | 0.01406 |
| 2 | 21,378,433 | chr2:21378433:D | I/D | 0.2230 | 0.93 (0.91-0.96) | 2.89E-08 | 21,378,778 | rs312984 | C/T | 0.1992 | 0.8523 | 345 | 0.94 (0.92-0.96) | 2.90E-07 | 1.02 (0.98-1.06) | 0.3848 |
| 3 | 138,099,161 | chr3:138099161:I | I/D | 0.2175 | 1.08 (1.05-1.1) | 2.89E-09 | 138,100,539 | rs1720819 | G/T | 0.2019 | 0.8800 | 1378 | 1.08 (1.05-1.11) | 5.06E-08 | 0.99 (0.94-1.04) | 0.7513 |
| 15 | 91,416,550 | rs17514846 | A/C | 0.4505 | 1.05 (1.03-1.07) | 3.10E-07 | 91,422,543 | rs8039305 | C/T | 0.4487 | 0.9473 | 5993 | 1.05 (1.03-1.07) | 1.45E-07 | 1.02 (0.99-1.06) | 0.2062 |
| 19 | 11,188,247 | rs56289821 | A/G | 0.0765 | 0.87 (0.85-0.9) | 4.44E-15 | 11,190,292 | rs112032422 | C/T | 0.0756 | 0.9642 | 2045 | 0.88 (0.85-0.91) | 2.29E-14 | 0.99 (0.94-1.04) | 0.6108 |
| 1 | 222,823,743 | rs67180937 | T/G | 0.4565 | 0.92 (0.9-0.94) | 1.01E-12 | 222,828,704 | rs17163363 | C/T | 0.4570 | 0.9985 | 4961 | 0.92 (0.9-0.94) | 1.23E-12 | 0.97 (0.93-1.01) | 0.09334 |
| 17 | 59,013,488 | rs7212798 | C/T | 0.2587 | 1.08 (1.05-1.11) | 1.88E-08 | 59,030,129 | rs1988961 | C/T | 0.2958 | 0.8320 | 16641 | 1.07 (1.04-1.1) | 2.87E-07 | 1.03 (0.99-1.07) | 0.1746 |

*bp: Base pair.

**eTable 3:** LD matrix of instrumental variables for 52 variants for CAD

|  | (chr1) rs11206510 | (chr1) rs17163363 | (chr1) rs6689306 | (chr1) rs7528419 | (chr1) rs9970807 |  |  |
| --- | --- | --- | --- | --- | --- | --- | --- |
| (chr1) rs11206510 | 1 | 0.001 | 0 | 0.01 | 0.002 |  |  |
| (chr1) rs17163363 |  | 1 | 0.004 | 0.008 | 0.003 |  |  |
| (chr1) rs6689306 |  |  | 1 | 0.004 | 0.003 |  |  |
| (chr1) rs7528419 |  |  |  | 1 | 0.003 |  |  |
| (chr1) rs9970807 |  |  |  |  | 1 |  |  |
|  |  |  |  |  |  |  |  |
|  | (chr2) rs17678683 | (chr2) rs312984 | (chr2) rs72932566 | (chr2) rs7568458 |  |  |  |
| (chr2) rs17678683 | 1 | 0.002 | 0.003 | 0.008 |  |  |  |
| (chr2) rs312984 |  | 1 | 0.001 | 0.018 |  |  |  |
| (chr2) rs72932566 |  |  | 1 | 0 |  |  |  |
| (chr2) rs7568458 |  |  |  | 1 |  |  |  |
|  |  |  |  |  |  |  |  |
|  | (chr4) rs17087335 | (chr4) rs4593108 | (chr4) rs72689147 |  |  |  |  |
| (chr4) rs17087335 | 1 | 0 | 0.002 |  |  |  |  |
| (chr4) rs4593108 |  | 1 | 0.016 |  |  |  |  |
| (chr4) rs72689147 |  |  | 1 |  |  |  |  |
|  |  |  |  |  |  |  |  |
|  | (chr6) rs12202017 | (chr6) rs17609940 | (chr6) rs4252185 | (chr6) rs55730499 | (chr6) rs56336142 | (chr6) rs6903956 | (chr6) rs9349379 |
| (chr6) rs12202017 | 1 | 0.001 | 0.002 | 0 | 0 | 0.005 | 0.013 |
| (chr6) rs17609940 |  | 1 | 0.001 | 0.003 | 0.013 | 0.003 | 0 |
| (chr6) rs4252185 |  |  | 1 | 0.438 | 0 | 0.002 | 0.001 |
| (chr6) rs55730499 |  |  |  | 1 | 0.001 | 0.005 | 0 |
| (chr6) rs56336142 |  |  |  |  | 1 | 0.001 | 0.011 |
| (chr6) rs6903956 |  |  |  |  |  | 1 | 0.024 |
| (chr6) rs9349379 |  |  |  |  |  |  | 1 |
|  |  |  |  |  |  |  |  |
|  | (chr7) rs10953541 | (chr7) rs11556924 | (chr7) rs2107595 | (chr7) rs3918226 |  |  |  |
| (chr7) rs10953541 | 1 | 0.009 | 0.002 | 0.012 |  |  |  |
| (chr7) rs11556924 |  | 1 | 0.01 | 0.024 |  |  |  |
| (chr7) rs2107595 |  |  | 1 | 0.002 |  |  |  |
| (chr7) rs3918226 |  |  |  | 1 |  |  |  |
|  |  |  |  |  |  |  |  |
|  | (chr8) rs264 | (chr8) rs2954029 |  |  |  |  |  |
| (chr8) rs264 | 1 | 0.013 |  |  |  |  |  |
| (chr8) rs2954029 |  | 1 |  |  |  |  |  |
|  |  |  |  |  |  |  |  |
|  | (chr9) rs2519093 | (chr9) rs2891168 |  |  |  |  |  |
| (chr9) rs2519093 | 1 | 0 |  |  |  |  |  |
| (chr9) rs2891168 |  | 1 |  |  |  |  |  |
|  |  |  |  |  |  |  |  |
|  | (chr10) rs11191416 | (chr10) rs1412444 | (chr10) rs1870634 | (chr10) rs2487928 |  |  |  |
| (chr10) rs11191416 | 1 | 0.009 | 0.019 | 0 |  |  |  |
| (chr10) rs1412444 |  | 1 | 0.006 | 0.001 |  |  |  |
| (chr10) rs1870634 |  |  | 1 | 0.004 |  |  |  |
| (chr10) rs2487928 |  |  |  | 1 |  |  |  |
|  |  |  |  |  |  |  |  |
|  | (chr11) rs10840293 | (chr11) rs2128739 | (chr11) rs964184 |  |  |  |  |
| (chr11) rs10840293 | 1 | 0.005 | 0 |  |  |  |  |
| (chr11) rs2128739 |  | 1 | 0.004 |  |  |  |  |
| (chr11) rs964184 |  |  | 1 |  |  |  |  |
|  |  |  |  |  |  |  |  |
|  | (chr12) rs2681472 | (chr12) rs3184504 |  |  |  |  |  |
| (chr12) rs2681472 | 1 | 0.021 |  |  |  |  |  |
| (chr12) rs3184504 |  | 1 |  |  |  |  |  |
|  |  |  |  |  |  |  |  |
|  | (chr13) rs11838776 | (chr13) rs9319428 |  |  |  |  |  |
| (chr13) rs11838776 | 1 | 0.001 |  |  |  |  |  |
| (chr13) rs9319428 |  | 1 |  |  |  |  |  |
|  |  |  |  |  |  |  |  |
|  | (chr15) rs4468572 | (chr15) rs56062135 | (chr15) rs8039305 | (chr15) rs8042271 |  |  |  |
| (chr15) rs4468572 | 1 | 0 | 0.001 | 0 |  |  |  |
| (chr15) rs56062135 |  | 1 | 0.001 | 0.018 |  |  |  |
| (chr15) rs8039305 |  |  | 1 | 0 |  |  |  |
| (chr15) rs8042271 |  |  |  | 1 |  |  |  |
|  |  |  |  |  |  |  |  |
|  | (chr17) rs12936587 | (chr17) rs1988961 | (chr17) rs216172 | (chr17) rs46522 |  |  |  |
| (chr17) rs12936587 | 1 | 0 | 0.003 | 0.003 |  |  |  |
| (chr17) rs1988961 |  | 1 | 0.004 | 0.104 |  |  |  |
| (chr17) rs216172 |  |  | 1 | 0.005 |  |  |  |
| (chr17) rs46522 |  |  |  | 1 |  |  |  |
|  |  |  |  |  |  |  |  |
|  | (chr19) rs112032422 | (chr19) rs4420638 |  |  |  |  |  |
| (chr19) rs112032422 | 1 | 0 |  |  |  |  |  |
| (chr19) rs4420638 |  | 1 |  |  |  |  |  |

**eTable 4**: Allele frequency of alleles at the *APOE* locus, by *APOE* SNPs

| **Allele**  **name** | **rs429358** | | |  | **rs7412** | | |
| --- | --- | --- | --- | --- | --- | --- | --- |
|  | **Base** | **Status** | **FRQ*** |  | **Base** | **Status** | **FRQ*** |
| ε2 | T | REF | 0.87 |  | T | OTHER | 0.07 |
| ε3 | T | REF | 0.87 |  | C | REF | 0.93 |
| ε4 | C | OTHER | 0.13 |  | C | REF | 0.93 |

* FRQ: Allele frequency taken from the CARDIOGRAM+C4D study.

**eTable 5:** Allele frequencies of additional alleles at the *APOE* locus, by *APOE* SNPs from CARDIoGRAM*plus*C4D

| **SNPs** | **A1** | **A2** | **A1 FREQ**  **(CARDIoGRAM*plus*C4D)** | **Description** |
| --- | --- | --- | --- | --- |
| rs6857 | C | T | 0.853 | IGAP peak SNP |
| rs429358 | T | C | 0.868 | *APOE* ε4 |
| rs7412 | C | T | 0.926 | *APOE* ε2 |
| rs4420638 | A | G | 0.834 | CARDIOGRAM peak SNP |

FRQ: Allele frequency.

**eTable 6:** Association of variants in the *APOE* locus with CAD, LOAD and LDL cholesterol concentrations

| **SNP** | **Description** | **RAF (CARDIOGRAM)** | **INFO (CARDIOGRAM)** | **CARDIOGRAM+C4D {1}** | | | | **IGAP {2}** | | | | **GLGC – LDL {3}** | | | |
| --- | --- | --- | --- | --- | --- | --- | --- | --- | --- | --- | --- | --- | --- | --- | --- |
|  |  |  |  | **beta** | **se** | **p-value** | **REF ALLELE** | **beta** | **Se** | **p-value** | **REF ALLELE** | **beta** | **se** | **p-value** | **REF ALLELE** |
| rs6857 | IGAP peak SNP | 0.85 | 0.83 | -0.08 | 0.01 | 6.83E-08 | C | 1.16 | 0.02 | 2.50E-575 | T | 0.19 | 0.01 | 5.12E-110 | T |
| rs429358 | *APOE ε4* allele | 0.87 | 0.89 | -0.09 | 0.02 | 2.17E-09 | T | 1.35 | 0.03 | 6.70E-536 | C | # | # | # | # |
| rs7412 | *APOE ε2* allele | 0.94 | 0.81 | 0.14 | 0.02 | 8.17E-11 | C | -0.39 | 0.04 | 1.23E-22 | T | 0.59 | 0.01 | 1.24E-652 | C |
| rs4420638 (rs41377151) | CARDIoGRAM*plus*C4D peak SNP | 0.83 | 0.88 | -0.09 | 0.01 | 7.07E-11 | A | 1.35 | 0.03 | 1.67E-396 | G | 0.23 | 0.01 | 1.51E-178 | G |

**eTable 7**: Pleiotropy at CAD variants using a Phenoscanner analysis of relevant traits

| **Variant** | **Locus** | **Alzheimer's**  **Disease** | **BMI** | **CRP** | **CAD** | **HDL** | **LDL** | **SBP** | **Total**  **Cholesterol** | **Triglycerides** | **Type II**  **diabetes** |
| --- | --- | --- | --- | --- | --- | --- | --- | --- | --- | --- | --- |
| rs10139550 | *HHIPL1* | NA | NA | NA | 1.38E-08 {1} | NA | NA | NA | NA | NA | NA |
| rs10840293 | *SWAP70* | NA | NA | NA | 1.28E-08 {1} | NA | NA | NA | NA | NA | NA |
| rs10953541 | *7q22* | NA | NA | NA | 3.12E-08 {4} | NA | 9.98E-05 {5} | NA | NA | NA | NA |
| rs11191416 | *CYP17A1-CNNM2-NT5C2* | NA | 1.55E-08 {6} | NA | 4.65E-09 {1} | NA | NA | 6.76E-10 {7} | NA | NA | NA |
| rs112032422 | *LDLR* | NA | NA | NA | 2.29E-14 {1} | NA | NA | NA | NA | NA | NA |
| rs11206510 | *PCSK9* | NA | NA | NA | 7.12E-41 {8} | NA | 2.38E-53 {3} | NA | 1.13E-41 {3} | 0.0002067 {9} | NA |
| rs11556924 | *ZC3HC1* | NA | NA | NA | 9.18E-18 {10} | 1.26E-05 {3} | NA | 0.000127 {7} | NA | NA | NA |
| rs11838776 | *COL4A1/A2* | NA | NA | NA | 1.83E-10 {1} | NA | NA | NA | NA | NA | NA |
| rs12202017 | *TCF21** | NA | NA | NA | 1.98E-11 {1} | NA | NA | NA | NA | NA | NA |
| rs12936587 | *RAI1-PEMT-RASD1* | NA | NA | NA | 2.00E-10 {11} | NA | NA | NA | NA | NA | NA |
| rs1412444 | *LIPA* | NA | NA | NA | 2.76E-13 {4} | NA | NA | NA | NA | NA | NA |
| rs17087335 | *REST-NOA1* | NA | NA | NA | 4.59E-08 {1} | NA | NA | NA | NA | NA | NA |
| rs17163363 | *MIA3* | NA | NA | NA | 1.23E-12 {1} | NA | NA | NA | NA | NA | NA |
| rs1720819 | *MRAS* | NA | NA | NA | 5.06E-08 {1} | NA | NA | NA | NA | NA | NA |
| rs17609940 | *ANKS1A* | NA | NA | NA | 1.36E-08 {10} | NA | NA | NA | NA | NA | NA |
| rs17678683 | *ZEB2-ACO74093.1* | NA | NA | NA | 3.00E-09 {1} | NA | NA | NA | NA | NA | NA |
| rs1870634 | *CXCL12* | NA | NA | NA | 5.55E-15 {1} | NA | NA | NA | NA | NA | NA |
| rs1988961 | *BCAS3* | NA | NA | NA | 2.87E-07 {1} | NA | NA | NA | NA | NA | NA |
| rs2107595 | *HDAC9* | NA | NA | NA | 3.00E-12 {11} | NA | NA | NA | NA | NA | NA |
| rs2128739 | *PDGFD* | NA | NA | NA | 7.05E-11 {1} | NA | NA | NA | NA | NA | NA |
| rs216172 | *SMG6* | NA | NA | NA | 1.15E-09 {10} | NA | NA | NA | NA | NA | NA |
| rs2487928 | *KIAA1462* | NA | NA | NA | 4.41E-11 {1} | NA | NA | NA | NA | NA | NA |
| rs2519093 | *ABO* | NA | NA | NA | 1.19E-11 {1} | NA | 1.25E-30 {3} | NA | 1.22E-26 {3} | NA | NA |
| rs264 | *LPL* | NA | NA | NA | 2.88E-09 {8} | 8.03E-77 {3} | NA | NA | 0.0007903 {12} | 2.35E-84 {3} | NA |
| rs2681472 | *ATP2B1* | NA | NA | NA | 6.17E-11 {1} | NA | NA | 5.00E-29 {13} | NA | NA | NA |
| rs273909 | *SLC22A4-SLC22A5* | NA | NA | NA | 9.62E-10 {8} | NA | 2.26E-05 {3} | NA | 0.0003691 {3} | NA | NA |
| rs28451064 | *KCNE2 (gene desert)* | NA | NA | NA | 1.33E-15 {1} | NA | NA | NA | NA | NA | NA |
| rs2891168 | *9p21* | NA | NA | NA | 2.29E-98 {1} | NA | NA | NA | NA | NA | NA |
| rs2954029 | *TRIB1* | NA | NA | NA | 4.75E-09 {8} | 2.67E-29 {3} | 2.00E-50 {3} | NA | 2.42E-65 {3} | 1.00E-107 {3} | NA |
| rs312984 | *APOB* | NA | NA | NA | 2.90E-07 {1} | NA | 3.15E-52 {3} | NA | 9.08E-45 {3} | NA | NA |
| rs3184504 | *SH2B3* | NA | 4.40E-06 {6} | NA | 1.74E-11 {8} | 4.10E-12 {3} | 4.20E-12 {3} | 3.80E-18 {7} | 1.62E-17 {3} | NA | NA |
| rs3918226 | *NOS3* | NA | NA | NA | 1.69E-09 {1} | NA | NA | 1.12E-06 {14} | NA | NA | NA |
| rs4252185 | *PLG* | NA | NA | NA | 1.64E-32 {1} | NA | NA | NA | NA | NA | NA |
| rs4420638 | *APOE-APOC1* | 1.67E-396 {2} | 5.30E-10 {15} | 8.80E-139 {16} | 7.07E-11 {1} | 2.30E-22 {9} | 1.51E-178 {3} | NA | 1.14E-149 {3} | 5.44E-22 {9} | 2.00E-07 {17} |
| rs4468572 | *ADAMTS7* | NA | NA | NA | 4.44E-16 {1} | NA | NA | NA | NA | NA | NA |
| rs4593108 | *EDNRA* | NA | NA | NA | 8.82E-10 {1} | NA | NA | NA | NA | NA | NA |
| rs46522 | *UBE2Z* | NA | NA | NA | 1.81E-08 {10} | NA | NA | NA | NA | NA | 0.0007272 {18} |
| rs55730499 | *SLC22A3-LPAL2-LPA* | NA | NA | NA | 5.39E-39 {1} | NA | NA | NA | NA | NA | NA |
| rs56062135 | *SMAD3* | NA | NA | NA | 4.52E-09 {1} | NA | NA | NA | NA | NA | NA |
| rs56336142 | *KCNK5* | NA | NA | NA | 1.85E-08 {1} | NA | NA | NA | NA | NA | NA |
| rs663129 | *PMAIP1-MC4R* | NA | 3.03E-57 {6} | NA | 3.20E-08 {1} | 5.54E-09 {3} | NA | NA | NA | 2.88E-05 {9} | 4.50E-08 {17} |
| rs6689306 | *IL6R* | NA | NA | NA | 2.60E-09 {1} | NA | NA | NA | NA | NA | NA |
| rs72689147 | *GUCY1A3* | NA | NA | NA | 6.07E-09 {1} | NA | NA | NA | NA | NA | NA |
| rs72932566 | *WDR12* | NA | NA | NA | 8.58E-18 {1} | NA | NA | NA | 0.0001683 {3} | NA | NA |
| rs7528419 | *SORT1* | NA | NA | NA | 1.97E-23 {1} | 9.55E-11 {3} | 1.55E-165 {3} | NA | 5.60E-110 {3} | NA | NA |
| rs7568458 | *VAMP5-VAMP8-GGCX* | NA | NA | NA | 3.62E-10 {1} | NA | NA | NA | NA | NA | NA |
| rs8039305 | *FURIN-FES* | NA | NA | NA | 1.45E-07 {1} | NA | NA | NA | NA | NA | NA |
| rs8042271 | *MFGE8-ABHD2* | NA | NA | NA | 3.68E-08 {1} | NA | NA | NA | NA | NA | NA |
| rs9319428 | *FLT1* | NA | 0.0006794 {6} | NA | 7.32E-11 {8} | NA | NA | NA | NA | NA | NA |
| rs9349379 | *PHACTR1* | NA | NA | NA | 1.81E-42 {1} | NA | NA | NA | NA | NA | NA |
| rs964184 | *ZNF259-APOA5-APOA1* | NA | NA | NA | 1.02E-17 {10} | 5.30E-50 {9} | 1.40E-27 {9} | NA | 1.30E-59 {9} | 7.60E-252 {9} | NA |
| rs9970807 | *PPAP2B* | NA | 0.0007588 {6} | NA | 5.00E-14 {1} | NA | NA | NA | NA | NA | NA |

**eTable 8:** Pleiotropic phenotypes at *APOE* peak variant rs6857 in IGAP

| **Phenotype (reference)** | **min p-value** |
| --- | --- |
| *Alzheimer’s Disease {2} | 2.50E-575 |
| ***LDL {3} | 5.12E-110 |
| ***Total cholesterol {3} | 1.70E-86 |
| *Neuritic plaque {19} | 3.00E-47 |
| *Neurofibrillary tangles {19} | 5.00E-47 |
| *Cerebral amyloid angiopathy {19} | 3.00E-21 |
| ***Triglycerides {9} | 4.28E-21 |
| ***HDL{9} | 5.51E-18 |
| **CAD {1} | 6.83E-08 |
| *Cortical amyloid beta load {20} | 1.06E-10 |

*Alzheimer's disease and related traits.

**CAD and related traits.

***Cholesterol and related traits.

**eTable 9:** Pleiotropic phenotypes at *APOE* peak variant rs4420638 in CARDIoGRAM*plus*C4D

| **Phenotype (reference)** | **min p-value** |
| --- | --- |
| *Alzheimer’s Disease {2} | 1.67E-396 |
| ***LDL {3} | 1.51E-178 |
| ***Total cholesterol {3} | 1.00E-149 |
| ****CRP {16} | 8.80E-139 |
| *Cognitive decline {21} | 4.00E-27 |
| ***HDL {9} | 2.30E-22 |
| ***Triglycerides {9} | 5.44E-22 |
| **CAD {1} | 7.07E-11 |

*Alzheimer's disease and related traits.

**CAD and related traits.

***Cholesterol and related traits.

****CRP.

**eTable 10:** Pleiotropic phenotypes at the variant rs429358 (*APOE* ε4) in Phenoscanner analysis of relevant traits

| **Phenotype (reference)** | **min p-value** |
| --- | --- |
| *Alzheimer’s Disease {2} | 6.70E-536 |
| *Cortical amyloid beta load {20} | 5.45E-14 |
| *Lewy body disease {19} | 1.00E-12 |
| *Cognitive ageing {22} | 3.66E-11 |
| ***LDL {23} | 4.21E-10 |
| **CAD {1} | 2.17E-09 |

*Alzheimer's disease and related traits.

**CAD and related traits.

***Cholesterol and related traits.

| **eTable 11:** Pleiotropic phenotypes at the variant rs7412 (*APOE* ε2) in Phenoscanner analysis of relevant traits |
| --- |

| **Phenotype (reference)** | **min p-value** |
| --- | --- |
| ***LDL {3} | 1.24E-652 |
| ***Total cholesterol {3} | 1.56E-283 |
| ***Lipid Metabolism {24} | 3.00E-58 |
| *Alzheimer’s Disease {2} | 1.23E-22 |
| ***Lipid traits {25} | 3.00E-53 |
| ***Triglycerides {3} | 1.15E-28 |
| ***HDL {3} | 4.44E-19 |
| **CAD {1} | 8.17E-11 |

*Alzheimer's disease and related traits.

**CAD and related traits.

***Cholesterol and related traits.

**eTable 12:** *Post-Hoc* power calculation for the GWS significant analysis and the FDR 214 analysis

| **Odds ratio** | ***Post-hoc* power (GWS)** | | ***Post-hoc* power (FDR 214)** | |
| --- | --- | --- | --- | --- |
|  | **se=0.0325** | **alpha=0.05** | **se=0.0230** | **alpha=0.05** |
| 1.05 | 0.3240 | | 0.5628 | |
| 1.07 | 0.5494 | | 0.8357 | |
| 1.08 | 0.6593 | | 0.9163 | |
| 1.10 | 0.8354 | | 0.9853 | |
| 1.15 | 0.9905 | | 1.0000 | |
| 1.20 | 0.9999 | | 1.0000 | |
| 1.30 | 1.0000 | | 1.0000 | |
| 1.50 | 1.0000 | | 1.0000 | |

**SUPPLEMENTARY REFERENCES**

1. CARDIoGRAM*plus*C4D Consortium. A comprehensive 1000 Genomes-based genome-wide association meta-analysis of coronary artery disease. *Nat Genet.* **47,** 1121-1130 (2015).

2. Lambert J.C. *et al*. Meta-analysis of 74,046 individuals identifies 11 new susceptibility loci for Alzheimer's disease. *Nat Genet* **45,** 1452-1458 (2013).

3. Global Lipids Genetics Consortium. Discovery and refinement of loci associated with lipid levels. *Nat Genet.* **45,** 1274-1283 (2013).

4. Coronary Artery Disease (C4D) Genetics Consortium. A genome-wide association study in Europeans and South Asians identifies five new loci for coronary artery disease. *Nat Genet.* **43,** 339-344 (2011).

5. Sabatti, C. *et al*. Genome-wide association analysis of metabolic traits in a birth cohort from a founder population. *Nat Genet.* **41,** 35-46 (2009).

6. Locke A.E. *et al.* Genetic studies of body mass index yield new insights for obesity biology. *Nature.* **518,** 197-206 (2015).

7. Ehret G.B. *et al*. Genetic variants in novel pathways influence blood pressure and cardiovascular disease risk. *Nature.* **478,** 103-109 (2011).

8. Deloukas P. *et al*. Large-scale association analysis identifies new risk loci for coronary artery disease. *Nat Genet.* **45,** 25-33 (2013).

9. Teslovich T.M. *et al*. Biological, clinical and population relevance of 95 loci for blood lipids. *Nature.* **466,** 707-713 (2010).

10. Schunkert H. *et al.* Large-scale association analysis identifies 13 new susceptibility loci for coronary artery disease. *Nat Genet.* **43,** 333-338 (2011).

11. Dichgans M. *et al.* Shared genetic susceptibility to ischemic stroke and coronary artery disease: a genome-wide analysis of common variants. *Stroke.* **45,** 24-36 (2014).

12. Barber M.J. *et al.* Genome-wide association of lipid-lowering response to statins in combined study populations. *PloS one.* **5,** e9763 (2010).

13. Kato N. *et al.* Meta-analysis of genome-wide association studies identifies common variants associated with blood pressure variation in east Asians. *Nat Genet.* **43,** 531-538 (2011).

14. Johnson T. *et al.* Blood pressure loci identified with a gene-centric array. *Am J Hum Genet.* **89,** 688-700 (2011).

15. Winkler T.W. *et al.* The Influence of Age and Sex on Genetic Associations with Adult Body Size and Shape: A Large-Scale Genome-Wide Interaction Study. *PLoS Genet.* **11,** e1005378 (2015).

16. Dehghan A. *et al.* Meta-analysis of genome-wide association studies in >80 000 subjects identifies multiple loci for C-reactive protein levels. *Circulation.* **123,** 731-738 (2011).

17. Mahajan A. *et al.* Genome-wide trans-ancestry meta-analysis provides insight into the genetic architecture of type 2 diabetes susceptibility. *Nat Genet.* **46,** 234-244 (2014).

18. Wellcome Trust Case Control Consortium. Genome-wide association study of 14,000 cases of seven common diseases and 3,000 shared controls. *Nature.* **447,** 661-678 (2007).

19. Beecham G.W. *et al.* Genome-wide association meta-analysis of neuropathologic features of Alzheimer's disease and related dementias. *PLoS Genet.* **10,** e1004606 (2014).

20. Ramanan V.K. *et al.* *APOE* and *BCHE* as modulators of cerebral amyloid deposition: a florbetapir PET genome-wide association study. *Mol Psychiatry.* **19,** 351-357 (2014).

21. De Jager P.L. *et al.* A genome-wide scan for common variants affecting the rate of age-related cognitive decline. *Neurobiol Aging.* **33,** 1017.e1011-1015 (2012).

22. Davies G. *et al.* A genome-wide association study implicates the *APOE* locus in nonpathological cognitive ageing. *Mol Psychiatry.* **19,** 76-87 (2014).

23. Thompson J.F. *et al.* Comprehensive whole-genome and candidate gene analysis for response to statin therapy in the Treating to New Targets (TNT) cohort. *Circ Cardiovasc Genet.* **2,** 173-181 (2009).

24. Kettunen J. *et al.* Genome-wide association study identifies multiple loci influencing human serum metabolite levels. *Nat Genet.* **44,** 269-276 (2012).

25. Wu Y. *et al*. Genetic association with lipids in Filipinos: waist circumference modifies an APOA5 effect on triglyceride levels. *J Lipid Res.* **54,** 3198-3205 (2013).

26. Wang F. *et al.* Genome-wide association identifies a susceptibility locus for coronary artery disease in the Chinese Han population. *Nat Genet.* **43**, 345-349 (2011).
